# Play fair: the impact of issue framing on attitudes toward transgender youth participation in sports

**DOI:** 10.3389/fspor.2026.1607634

**Published:** 2026-04-08

**Authors:** Donald P. Haider-Markel, Andrew R. Flores, Daniel C. Lewis, Patrick R. Miller, Jami K. Taylor

**Affiliations:** 1 University of Kansas, Lawrence, KS, United States; 2 American University, Washington, DC, United States; 3 Siena College, Colonie, NY, United States; 4 Kent State University, Kent, OH, United States; 5 University of Toledo, Toledo, OH, United States

**Keywords:** attitudes, framing, policy, sports, transgender

## Abstract

**Introduction:**

Policies restricting sports participation by transgender youth have spread across the United States. The Trump administration has also taken a variety of executive actions to ban participation by transgender athletes.

**Methods:**

Little research has examined how issue framing affects attitudes toward these policies. Some LGBT advocates recommend emphasizing egalitarian and loss-based arguments, as well as acknowledging discomfort regarding transgender youth. We investigate whether these strategies affect Americans’ attitudes on transgender participation in sports.

**Results:**

Using a survey sample of American adults, we find that egalitarian frames have a marginal effect on respondents’ attitudes.

## Introduction

More than half of the American states have passed laws restricting sports participation by transgender youth (1).^1^ In addition, the Trump administration has taken actions such as executive orders and… to block transgender women and girls from competing in women's sports at all educational institutions covered by Title IX (2). In general, these bans have been pushed by conservatives for the electoral advantage of Republicans (3). Indeed, the issue of transgender participation in sports was extensively used by Republican candidates during the 2024 election cycle (4, 5). This strategy was arguably effective as an electoral tool because there is no strong public support for transgender participation in sports (6), and transgender rights have broadly become more partisan over time (7, 8). To counter these trends, transgender advocates need to change public attitudes toward transgender participation in sports.

Using a preregistered experiment, we examine activist-recommended communication strategies regarding transgender youth participation in sports (9). These strategies include the deployment of egalitarian or loss-based frames and acknowledging that some people may feel discomfort with transgender people. We find that utilizing egalitarian frames can slightly influence attitudes toward transgender youth participation in sports.

## Public attitudes toward transgender rights and transgender people

Research on public attitudes toward transgender rights shows that a portion of the public feels uncomfortable with transgender people and tends to view them less warmly than other minority groups (10). However, public support varies across different transgender-related issues (11). For instance, there is greater support for traditional civil rights concerns (11).

However, issues involving transgender bodies, such as restroom access or sports participation, generate greater public opposition (11), especially among Republicans and conservatives (7). Individuals who are more religious also tend to oppose transgender rights (12, 13). In contrast, women and those having interpersonal contact with members of the LGBT community are more supportive of transgender rights (14). Personality traits and dispositions, such as authoritarianism, moral traditionalism, and disgust sensitivity, are negatively associated with support for transgender rights and comfort with transgender people (10, 13). Conversely, egalitarianism, a concept rooted in principles of equality and fairness, is positively associated with support for transgender rights (6, 15). Finally, we know that partisan elite messaging shapes public attitudes toward transgender rights (7).

## Framing transgender rights

Political actors seek to shape public opinion by providing cueing and persuasive messages (16). Cueing messages provide contextual information about the political implications of an issue, whereas persuasive messages present arguments that support a position. These arguments are framed in ways that influence how individuals perceive issues (17, 18). For instance, by providing cues, values-based messages can reframe policy discussions and facilitate changes in public opinion (19). People are also more persuaded by arguments that emphasize the losses incurred by a policy (20).

We know that every issue has more than one dimension. The issue framing literature suggests that invoking one dimension of an issue over others can persuade the audience to consider that dimension over others. This frame can then shape subsequent attitudes. For example, if a speaker discussing gun policy frames the issue in terms of public safety, that frame becomes more accessible in the mind of listeners, and the audience member may be more likely to consider public safety when considering the regulation of firearms (21).

In LGBT rights policy discourse, proponents often deploy equality-based frames to shape public attitudes (22), while opponents often emphasize morality and traditional values (22). Tadlock (23) examined frames for transgender rights and found substantial overlap between the framing of gay and transgender rights, suggesting that equality and morality frames both dominate both discourses. In addition to equality frames, education frames are also important in transgender rights advocacy. This framing helps the public understand transgender people and transgender identity, given the lack of contact and greater discomfort that people have with transgender individuals (10). Relatedly, acknowledging personal discomfort with transgender identity may increase receptivity to persuasive messages (24).

We contend that value activation occurs when specific issue frames make certain normative considerations more cognitively accessible, thereby guiding how individuals evaluate policies related to transgender youth participation in sports. In this case, any given policy issue has multiple potential dimensions, and framing works by highlighting one dimension—such as equality or loss—so people rely more on that value when forming opinions. An egalitarian frame, for example, explicitly cues concerns about fairness and discrimination (“we shouldn't discriminate against kids and ban them from playing because they're transgender”), which activates egalitarian predispositions and leads some respondents—especially those with mixed egalitarian and inegalitarian values—to interpret bans as unequal treatment, rather than as a matter of competitive fairness or moral order. In contrast, the loss frame attempts to activate empathic concern by stressing the developmental and psychological harms that follow from excluding transgender youth from sports. However, respondents who lack strong empathic bonds with transgender people are unlikely to change their views. We also know that motivated reasoning will likely dampen the impact of such appeals. Thus, the psychological mechanism of value activation in this context is a selective accessibility process, in which frames highlight particular values (equality vs. harm/loss), and these activated values then shape attitude formation, with egalitarian cues perhaps increasing support for inclusion of transgender youth in sports.

The Movement Advancement Project (9), a pro-LGBT think tank, recommends best practices for communicating about transgender youth participation in sports, including equality and loss framing and acknowledging discomfort as useful approaches. We concur and argue that such frames activate particular values and make them accessible to respondents. Therefore, we propose the following hypotheses:

- H1: Acknowledging discomfort will positively influence attitudes toward transgender sports bans.
- H2: Loss frames will positively influence attitudes toward transgender sports bans.
- H3: Egalitarian frames will positively influence attitudes toward transgender sports bans.
- H4: Acknowledging discomfort will increase framing effects.

## Method

### Participants

We conducted a survey experiment between 14 and 16 March 2023, using Lucid Theorem, an online survey platform, funded by the Williams Institute at UCLA. Initially, 10,988 adult participants were recruited via email and provided a link to a Qualtrics-based survey. With approval from the Institutional Review Boards of American University and the University of Toledo, participants were presented with informed consent and confirmed that they were adults. Respondents who consented, completed the questionnaire, and passed two attention checks were returned to Lucid as complete cases (*n* = 3,694). Lucid then provided compensation to these participants. Supplementary Appendix A1 includes our survey questions. Participants were 59% female, 73% White, 55% college graduates, 29% strongly democratic, 11% independent, and 14% strongly republican, with an average age of 49.7 years (SD = 16.3).

### Pretest

Much of the respondents’ demographic information was preloaded into the Lucid panel, including gender, partisanship, age, household income, race and ethnicity, ZIP code, region, and education. The pretest measured ideology, religiosity, born-again identification, and interest in sports (termed “sports fandom”). Ideology was measured using a self-placed seven-point scale, where lower values indicated more liberal views and higher values indicated more conservative views. The frequency of religious service attendance was measured using a six-point scale ranging from more than once per week to never. We also asked whether individuals considered themselves born again. Sports fandom was measured on a 0–10 scale, with higher values indicating greater interest in sports (6).

Subsequently, each respondent was given an informational vignette about transgender identity:

“Gender identity refers to how a person identifies their own gender (as a man, woman, or some other label). For many people their gender identity may not match their birth sex.

For example, a male may identify more as a woman, or a female may identify more as a man. Transgender is a general term for people whose gender identity or expression is different from their birth sex.

When we say the term transgender girl, we mean someone whose birth sex was male, but they identify as a girl.

When we say the term transgender boy, we mean someone whose birth sex was female, but they identify as a boy.”

Afterward, we included a dichotomous question about LGBT self-identification. Contact with gay, lesbian, bisexual, and transgender people was assessed, with response options differentiating whether contact was with family members, close friends, acquaintances, or there was no contact. Respondents were allowed to mark all types of contact.

The pretest also measured several psychological traits, including authoritarianism, moral traditionalism, gender role beliefs, disgust sensitivity, egalitarianism, and Christian nationalism. Authoritarianism was measured using four ranked-choice items from the American National Election Studies (25), and an additive scale was constructed, with higher values indicating greater authoritarianism in child-rearing (*α* = 0.66). Egalitarianism and moral traditionalism were measured using four five-point Likert-scale items (25), and additive scales were constructed, with higher values indicating greater egalitarianism (*α* = 0.64) and stronger moral traditionalism (*α* = 0.71). Gender role traditionalism was measured using four five-point Likert-scale items from Kerr and Holden (40), with higher values indicating greater gender role traditionalism (*α* = 0.76). Disgust sensitivity was measured using three items from Haidt et al. (26), with higher values indicating greater disgust sensitivity on this scale (*α* = 0.71). Christian nationalism was measured using four items from the Baylor Religion Survey (27), with higher values representing greater Christian Nationalism on this scale (*α* = 0.76). Finally, comfort with transgender people was assessed using a question with a four-point response set ranging from comfortable to uncomfortable (10).

All scales were standardized to have a mean of zero and a standard deviation of one. For any item missingness on pretest scales, respondents were mean-imputed to reduce pretest non-response bias, following recommended practices (28) and preregistration. Any pretest item missingness on ordinal measures was median-imputed, which occurred for one observation on church attendance and comfort with transgender people.

Table 1 presents summary statistics of demographics and pretest measures. While the sample was drawn from an opt-in panel, it remained demographically diverse, though the respondents were somewhat politically progressive.

**Table 1:** Summary statistics of demographics and pretest measures.

Variable	Mean	SD	Min	Max
Female	0.59	0.49	0	1
Age	49.7	16.3	18	95
Race/ethnicity				
White	0.73	0.45	0	1
Black	0.09	0.28	0	1
Latino	0.09	0.29	0	1
Other	0.09	0.29	0	1
College graduate	0.55	0.50	0	1
Partisanship (D → R)	2.50	2.15	0	6
Strong democrat	0.29	0.46	0	1
Weak democrat	0.10	0.29	0	1
Lean democrat	0.12	0.32	0	1
Independent	0.17	0.37	0	1
Lean republican	0.11	0.31	0	1
Weak republican	0.08	0.27	0	1
Strong republican	0.14	0.35	0	1
Ideology (Lib. → Con.)	2.93	1.86	0	6
Very liberal	0.14	0.34	0	1
Liberal	0.13	0.34	0	1
Somewhat liberal	0.08	0.27	0	1
Moderate	0.30	0.46	0	1
Somewhat conservative	0.10	0.30	0	1
Conservative	0.14	0.34	0	1
Very conservative	0.11	0.31	0	1
Church attendance	2.03	1.82	0	5
More than once a week	0.29	0.46	0	1
Once a week	0.20	0.40	0	1
Once or twice a month	0.13	0.33	0	1
A few times a year	0.07	0.26	0	1
Seldom	0.17	0.38	0	1
Never	0.13	0.34	0	1
Born again	0.37	0.48	0	1
LGBT	0.16	0.37	0	1
Gay contact	0.68	0.47	0	1
Transgender contact	0.29	0.45	0	1
Bisexual contact	0.38	0.48	0	1
Sports fan	5.83	3.43	0	10
Not a fan at all (0)	0.12	0.32	0	1
1	0.07	0.25	0	1
2	0.06	0.24	0	1
3	0.04	0.20	0	1
4	0.04	0.18	0	1
5	0.08	0.27	0	1
6	0.06	0.24	0	1
7	0.12	0.32	0	1
8	0.13	0.34	0	1
9	0.13	0.34	0	1
Super sports fan (10)	0.15	0.36	0	1
Authoritarianism	0.00	1.00	−1.54	1.38
Moral traditionalism	0.00	1.00	−2.47	1.93
Gender roles	0.00	1.00	−2.05	2.01
Disgust sensitivity	0.00	1.00	−1.65	1.71
Egalitarianism	0.00	1.00	−1.71	2.96
Christian nationalism	0.00	1.00	−2.14	1.60

### Experimental design

Respondents were randomly divided into groups in a 2 (discomfort)×3 (frame) factorial design, in which respondents read vignettes that either acknowledged discomfort or did not, and provided one of three message types: egalitarian framing, loss framing, or no framing. Table 2 presents the text of the vignettes and the number of respondents in each condition. The vignette text was drawn from the communications guide of the Movement Advancement Project (9) on transgender youth and sports.

**Table 2:** Treatment vignettes and sample sizes.

	Acknowledge discomfort condition		Total
Frame condition	Control	Acknowledge discomfort	
Control	“Press the arrow to continue the survey”	“It can be hard to understand what it means to be transgender, especially if you’ve never met a transgender person. And it's common to have questions at first. Transgender youth grow up knowing deep down that their sex at birth doesn't match who they know they are inside.”	1,233 (33%)
	(*n* = 615)	(*n* = 618)	
Egalitarian	“Transgender kids want the opportunity to play sports for the same reasons other kids do: to be a part of a team where they feel like they belong. We shouldn't discriminate against kids and ban them from playing because they're transgender.”		1,230 (33%)
	(*n* = 614)	(*n* = 617)	
Loss	“Participation in youth sports improves physical wellbeing and it builds confidence, self-respect, leadership and teamwork skills. Student athletes are less likely to engage in risky behaviors like drug use, dropping out of school, and resorting to self-harm. They are more likely to excel academically and take on leadership positions as adults. Bans on transgender participation in youth sports take away these vital opportunities from these at-risk kids. Taking away youth sports from transgender children will make their lives even more challenging than they already are.”		1,231 (33%)
	(*n* = 609)	(*n* = 621)	
Total	1,838 (49.8%)	1,856 (50.2%)	3,694

### Post-test

In the post-test, we asked a battery of 19 items related to transgender athletes and sports. Using a four-point response set, we asked:

- Do you agree or disagree that transgender youth should only be allowed to participate in K-12 sports based on their sex assigned at birth?

We also asked respondents to indicate their level of agreement or disagreement with the following two statements (four-point response set):

- Transgender girls should be banned from participating in K-12 girls’ sports.
- Transgender boys should be banned from participating in K-12 boys’ sports.

Respondents were also asked whether transgender women and girls should be allowed to compete at different levels of athletics: professional sports, college sports, high school sports, and youth sports. The response options for each level were: should be allowed, not be allowed, and no opinion. Given the distinction between opposition to a practice and government action banning it, we asked about state bans using the following items:

- State laws should ban transgender girls from competing against other girls in youth sports.
- State laws should ban transgender women from competing against other women in college sports.

Each item used a five-point response set. We asked respondents to state their agreement or disagreement with 10 statements regarding transgender youth and sports (see Supplementary Appendix A1 for full wording of the questions). Finally, we included a feeling thermometer for transgender people and other groups.

### Analysis

**Figure 1:**
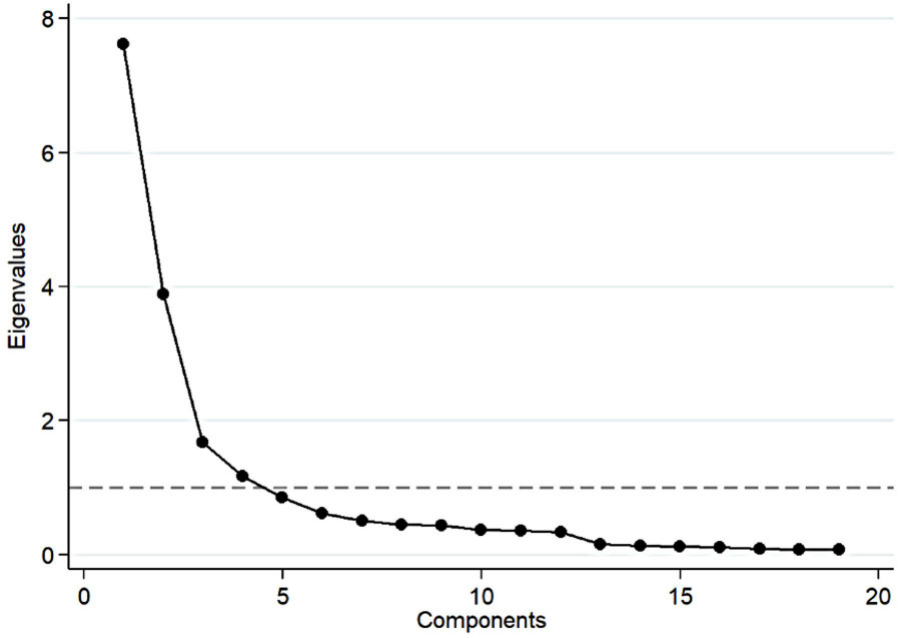
Scree plot.

As preregistered, we focused on the first policy question, which asked whether transgender youth should be allowed to compete in sports based on their sex assigned at birth, and we constructed a scale (transgender sport scale) using a conventional principal component analysis (PCA) of all 19 sports-related items.^2^ The eigenvalues are reported in Supplementary Appendix A1, and the scree plot is shown in Figure 1. Notably, the first component explained 40.1% of the total variance, and only the first four components had eigenvalues greater than one.

We did not preregister any analyses for the feeling thermometers. Since item missingness was minimal for the first policy question (*n* = 3) or for the standardized scale (*n* = 101), we present complete-case analyses, along with Horowitz–Manski worst-case bounds (29). Although missingness was not an issue (five cases), using imputation kept us consistent with the strategy listed in our preregistration. The small number of cases did not substantively influence our results.

Before analyzing the treatment effects, we used demographic variables and pretest measures to identify the variables that best predicted the two outcomes, thereby improving analytical efficiency (30). In our preregistration document, we provided power analyses showing the efficiency gains for both the main and interactive effects. Based on prior studies on opinions about transgender rights, we considered that our pretest measures would explain about 35% of the variation in the dependent variables. To select variables for the analysis, a robust LASSO machine learning algorithm was applied to each dependent variable. Ordinary least squares models were used to estimate both main and interactive treatment effects, with heteroskedasticity-consistent robust standard errors (HC3) (31, 32). We also presented topline results by treatment arm to initially summarize findings.^3^ Additional results are documented in the Supplementary Appendix.

## Results

Table 3 presents the results for the two dependent variables by treatment status. A large majority of respondents strongly or somewhat agreed with policies that would require transgender youth to participate in sports based on their sex assigned at birth. Although opinions did not differ much between the control group and the loss-frame group, there was a reduction in strong agreement and an increase in strong disagreement with this policy proposal among those exposed to the egalitarian frame. A test of independence indicated an association between frame assignment and agreement with the policy. Attitudes toward this policy did not differ statistically between respondents whose discomfort was acknowledged and those whose discomfort was not acknowledged.

**Table 3:** Responses to opinions about sports participation based on sex assigned at birth and the transgender sports scale, presented by treatment groups.

Responses	DV: Transgender youth should only be allowed to participate in K-12 sports based on their sex assigned at birth				
	Frame condition			Discomfort condition	
	Control	Loss frame	Egalitarian frame	Control	Acknowledge discomfort
	% (*n*)	% (*n*)	% (*n*)	% (*n*)	% (*n*)
Strongly agree	41.6 (513)	40.5 (497)	36.4 (448)	40.2 (738)	38.8 (720)
Somewhat agree	24.2 (298)	24.1 (296)	27.2 (335)	24.2 (444)	26.1 (485)
Somewhat disagree	20.5 (252)	18.6 (228)	19.1 (235)	19.5 (358)	19.2 (357)
Strongly disagree	13.7 (169)	16.9 (207)	17.3 (213)	16.1 (295)	15.8 (294)
*N*	1,232	1,228	1,231	1,835	1,856
*χ*^2^ (*df*)	14.9 (6)*	1.9 (3)			
	DV = Transgender sports scale				
	Frame condition			Discomfort condition	
	Control	Loss frame	Egalitarian frame	Control	Acknowledge discomfort
	*M* (SD)	*M* (SD)	*M* (SD)	*M* (SD)	*M* (SD)
	−0.05 (1.00)	−0.002 (0.99)	0.05 (1.01)*	−0.001 (0.99)	0.001 (1.01)

Pearson's chi-square test of independence is used for the categorical dependent variable and two-sample *t*-tests for differences in means between treatment and control conditions; *p < 0.05.

Similarly, the average scores on the transgender sports scale were slightly and significantly higher among respondents in the egalitarian frame condition relative to those in other conditions. However, average responses did not differ between the control group and those in the loss frame condition, and average responses did not differ between respondents whose discomfort was acknowledged and those whose discomfort was not acknowledged.

**Figure 2:**
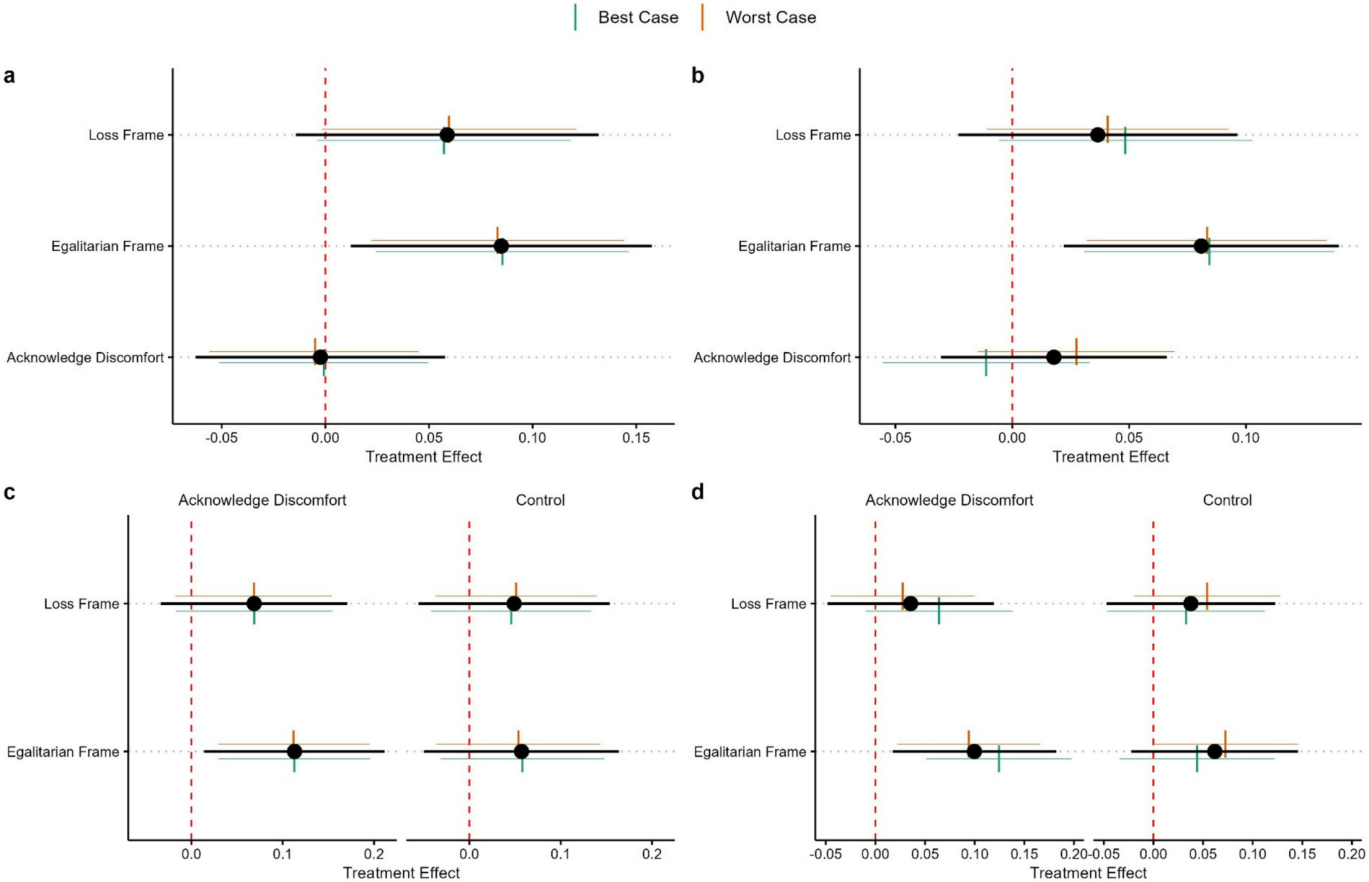
Regression-adjusted results showing **(a)** average treatment effects on opinions about sports participation based on sex assigned at birth; **(b)** average treatment effects on the transgender sports scale; **(c)** conditional average treatment effects of the frame by acknowledging discomfort on opinions about sports participation based on sex assigned at birth; and **(d)** conditional average treatment effects of the frame by acknowledging discomfort on the transgender sports scale. In the figure, 95% confidence intervals are plotted around the treatment effects, while 90% confidence intervals are plotted around the Horowitz–Manski worst-case bounds.

Figure 2 presents the regression-adjusted treatment effect estimates, along with estimates of Manski–Horowitz worst-case bounds. For the policy about sex assigned at birth, the models controlled for age, partisanship, egalitarianism, disgust, gender roles, traditionalism, church attendance, born-again identification, and sports fandom and had an *R*^2^ = 0.29 before assessing treatment effects. For the transgender sports scale, the models controlled for gender, partisanship, egalitarianism, traditionalism, comfort, ideology, and sports fandom and had an *R*^2^ = 0.46 before assessing treatment effects.

Figures 2a,b present the regression-adjusted average treatment effects on the policy and the transgender sports scale. For both dependent variables, we found that the egalitarianism frame increased the likelihood of a more favorable stance toward transgender participation in sports. However, the effects were not large. For the policy question, opinions were about 0.08 points higher in the egalitarian frame condition than in the control condition. On the transgender sports policy scale, opinions were about 0.08 SDs higher in the egalitarian condition relative to the control. We also did not observe significant treatment effects for the loss framing condition or the acknowledging discomfort condition on either dependent variable. We further found that the worst-case bounds and their confidence intervals did not differ much for any average treatment effect estimate, suggesting that our findings were not highly sensitive to missing post-test observations. Thus, we are confident that egalitarian framing changed opinions to be more supportive of transgender youth participation in sports, although the effect size was small.

Our preanalysis plan suggested that we may be underpowered to detect fully interactive effects, and we did not find a statistically significant interaction between the frame arm and the acknowledge discomfort arm of our experiment. However, our findings were adequately powered to investigate conditional average treatment effects of the frame arm depending on whether discomfort was acknowledged. Figures 2c,d plot these conditional average treatment effects for the policy concerning participation based on sex assigned at birth and for the transgender sports scale. The loss frame did not produce statistically significant treatment effects relative to the control frame, regardless of whether discomfort was acknowledged, for either dependent variable. Interestingly, we found that the egalitarian frame produced a statistically significant effect only when discomfort was acknowledged, while the effect was not statistically significant when discomfort was not acknowledged.^4^

## Discussion

The rapid diffusion of policies restricting transgender participation in school athletics is not unexpected. Under the right conditions, such morality policies can spread quickly (33). In this case, many states operate under unified Republican control, and nationally networked activists are working to spread these policies (34, 35). The American public's position on this topic appears to be ambivalent at best (6). Because of this ambivalence, political elites can shape public attitudes (7). It is in this context that we examined issue frames that may be used by LGBT activists interested in combating policies that ban transgender participation in sports.

We found that acknowledging discomfort with transgender identity or deploying loss frames was not noticeably effective in shaping attitudes toward transgender people participation in sports. Thus, H1 and H2 were not supported. This result was surprising, as previous research has reported a significant impact of acknowledging discomfort (24). If one considers acknowledging discomfort as an opportunity to provide insight into transgender lives, it can function as an education frame, which has long been a component of transgender rights advocacy (23).

Perhaps the increasing salience of transgender rights, combined with the increasing partisan divide over transgender rights, has decreased the need for this type of education framing (7); people are increasingly familiar with this topic. However, there is a difference between familiarity and being knowledgeable. We suspect that, although more Americans have become familiar with the topic of transgender people, this familiarity may still reflect relatively shallow knowledge. We also suspect that the lack of value activation may have resulted from low empathy toward transgender people or from motivated reasoning by participants that prevented value activation.

It is possible that our efforts to measure discomfort or use a loss frame were not powerful enough to influence participants’ attitudes. However, given the strong role of motivated reasoning among those who are less comfortable with transgender people, attempts to shift these attitudes are difficult (24).

Interestingly, H3 was supported. We found that deployment of an egalitarian frame could provide a small shift in respondents’ attitudes regarding sports participation by transgender people. This framing is consistent with longtime frames used by LGBT advocates (22, 23). Egalitarian framing may help people contextualize the issues in terms of values related to equality and discrimination. For instance, our heterogeneous effects analysis of the egalitarian frame by respondent egalitarianism suggests that individuals with a mix of egalitarian and inegalitarian values are more persuaded by the egalitarian frame. Their predispositions and the linkage of LGBT rights with egalitarian appeals may make this subset more ready to shift their attitudes; whereas high egalitarians are already more approving, low egalitarians are already less approving.

Finally, we found very limited support for H4. There were no interactive effects between acknowledging discomfort and the loss framing condition on attitudes toward transgender participation in sports.

However, an interactive effect was observed between acknowledging discomfort and the deployment of the egalitarian frame on both dependent variables, although not to a degree that we can say that the effect of the egalitarian frame was significantly different when acknowledging discomfort than not.

The effectiveness of the egalitarian frame may be rooted in classical arguments for LGBT rights (22, 23). The failure of the loss frame may stem from low empathy toward transgender youth. Prior research by Broockman and Kalla (36) shows that increasing empathy for transgender people may change opinions, suggesting that empathy is low. The loss frame emphasized that transgender youth may miss out on many of the benefits of sports while also suggesting that transgender kids were already “at-risk.” Perhaps that treatment requires stronger empathic bonds with transgender people; however, Flores et al. (37) provided little evidence in other areas of transgender rights attitudes that interventions to increase empathy enhanced the effectiveness of arguments favoring transgender rights.

Our study has several limitations. First, our study may have been underpowered to investigate interactive effects. Second, our study design did not address how countervailing messages might affect respondent attitudes. This is important because conservative advocacy groups and Republican candidates actively communicate on this topic (4, 5, 38). Given that the sample was largely opposed to transgender inclusion in sports, conservative messaging may be working. Further, the nature of our sample limits the generalizability of our findings to the larger population. Finally, our study is limited by the fact that we were unable to conduct a second-wave survey to assess the durability of our treatments over time.

In addition, although we asked respondents whether they identified as LGBTQ, we did not ask about specific identities within this category. Although the number of respondents is likely to be small, future studies should try to better account for LGBTQ status. Likewise, because most state policies restricting transgender athletes focus on sex assigned at birth rather than whether an athlete is male or female, our questions about transgender athletes did not make a distinction. Although Republican messaging on transgender athletes has focused on transgender girls and women, the enforcement of the laws in places such as Texas has also applied to transgender boys (39). Future research should examine whether respondents respond differently to transgender athletes depending on whether they identify as male or female. We suspect that transgender boys are generally not the “problem,” only transgender girls.

Despite these limitations, our work provides useful insights for those advocating in this policy space. Egalitarian framing remains a useful tool for groups advocating for LGBT rights.

## Data Availability

The raw data supporting the conclusions of this article will be made available by the authors, without undue reservation.

## Footnotes

^1^Gender identity refers to how a person identifies their own gender (identifying as a man, woman, or another label). For many people, gender identity does not align with their birth sex. For example, a man may identify more as female or a woman may identify more as male. Transgender is a general term used to describe individuals whose gender identity or expression differs from their birth sex. Some transgender people may undergo gender transition with medical procedures, such as hormone therapy or surgery (often commonly called a “sex change”), while others may not seek such medical assistance. The term transgender is often used as an umbrella term that can include many groups, including transsexuals, cross-dressers, or genderqueer, or gender non-conforming people.

^2^We standardized the first component.

^3^Initial summary analyses were not pre-registered.

^4^As noted, the full interaction of the frame arm and the acknowledge discomfort arm was not statistically significant and might be underpowered. Thus, the differences in conditional average treatment effects were not statistically significant for the policy (*Δ* = 0.06, se = 0.07, *p* = 0.46) or the scale (*Δ* = 0.04, *se* = 0.06, *p* = 0.53).
