# Supplementary material for: Play fair: the impact of issue framing on attitudes toward transgender youth participation in sports
Source: Front Sports Act Living. 2026 Apr 8;8:1607634. doi: 10.3389/fspor.2026.1607634 (PMC13099774; doi:10.3389/fspor.2026.1607634)
Supplement: Supplementary file 1 [file Datasheet1.pdf]

Appendix A: Survey Questions

Authoritarianism Scale
Which one is more important for a child to have? A child should have:

a. Independence or respect for elders
b. Obedience or self-reliance
c. Curiosity or good manners
d. Being considerate or well behaved.

RESPONSE CATEGORIES (select one option)

e. Independence or respect for elders
f. Obedience or self-reliance
g. Curiosity or good manners
h. Being considerate or well behaved.

Christian Nationalism Scale
How much do you agree or disagree with the following statements [Randomize a-d]?

a. The federal government should declare the United States a Christian nation.
b. The federal government should advocate Christian values.
c. The federal government should enforce strict separation of church and state.
d. The success of the United States is part of God's plan.

RESPONSE CATEGORIES

a. Strongly agree
b. Somewhat agree
c. Neither agree nor disagree
d. Somewhat disagree
e. Strongly disagree

Comfort
Are you, personally, comfortable or uncomfortable around someone who is transgender?

RESPONSE CATEGORIES

a. Comfortable
b. Somewhat comfortable
c. Somewhat uncomfortable
d. Uncomfortable

Contact
Now thinking about the people that you know; do you know someone who is: (select all that apply)

a. Gay or lesbian
b. Bisexual
c. Transgender

RESPONSE CATEGORIES

a. Yes, a family member
b. Yes, a close friend
c. Yes, an acquaintance such as a co-worker or someone from school or college.
d. No

Disgust Sensitivity Scale
Please tell us how the following statements describe you.

a. I never let any part of my body touch the toilet seat in a public washroom.
b. I probably would not go to my favorite restaurant if I found out that the cook had a cold.
c. I use hand sanitizer on a daily basis.

Egalitarianism Scale
Below is a series of statements about contemporary society. Please indicate the degree to which you agree or disagree with each statement. [Randomize a-d]

a. This country would be better off if we worried less about how equal people are.
b. It is not really that big a problem if some people have more of a chance in life than others.
c. If people were treated more equally in this country, we would have many fewer problems.
d. Our society should do whatever is necessary to make sure that everyone has an equal opportunity to succeed.

RESPONSE CATEGORIES

f. Strongly agree
g. Somewhat agree
h. Neither agree nor disagree
i. Somewhat disagree
j. Strongly disagree

Evangelical
Do you consider yourself to be Born Again?

RESPONSE CATEGORIES

a. No
b. Yes

Ideology
Where would you place yourself on this scale, or haven't you thought much about this?

RESPONSE CATEGORIES

a. Extremely liberal
b. Liberal
c. Slightly liberal
d. Moderate: middle of the road
e. Slightly conservative
f. Conservative

g. Extremely conservative

LGBT Identification
Do you, personally identify as lesbian, gay bisexual, or transgender?

RESPONSE CATEGORIES
a. No
b. Yes

Moral Traditionalism Scale
Below is a series of statements about contemporary society. Please indicate the degree to which you agree or disagree with each statement [Randomize a-d].
a. The world is always changing and we should adjust our view of moral behavior to those changes.
b. The newer lifestyles are contributing to the breakdown of our society.
c. We should be more tolerant of people who choose to live according to their own moral standards, even if they are very different from our own.
d. This country would have many fewer problems if there were more emphasis on traditional family ties.

RESPONSE CATEGORIES:
a. Strongly agree
b. Somewhat agree
c. Neither agree nor disagree
d. Somewhat disagree
e. Strongly disagree

Religious Service Attendance
Lots of things come up that keep people from attending religious services even if they want to. Aside from weddings and funerals, how often do you attend religious services?

RESPONSE CATEGORIES
a. More than once a week
b. Once a week
c. Once or twice a month
d. A few times a year
e. Seldom
f. Never

Sports Fan Identification
On a scale from 0 to 10, with 0 being no interest and 10 being a strong interest, how strong of a sports fan are you?

Dependent Variables
Do you agree or disagree that transgender youth should be only allowed to participate in K-12 sports based on their sex at birth?

RESPONSE CATEGORIES
a. Strongly agree
b, Somewhat agree
c. Somewhat disagree
d. Strongly disagree

Dependent variable: Transgender Sports Scale

Do you agree or disagree that transgender youth should be only allowed to participate in K-12 sports based on their sex at birth?

RESPONSE CATEGORIES
a. Strongly agree
b, Somewhat agree
c. Somewhat disagree
d. Strongly disagree

Please tell us how much you agree or disagree with each statement.
a. Transgender girls should be banned from participating in K-12 girls' sports.
b, Transgender boys should be banned from participating in K-12 boys' sports.

RESPONSE CATEGORIES
a. Strongly agree
b, Somewhat agree
c. Somewhat disagree
d. Strongly disagree

Thinking about transgender women and girls, do you think transgender women and girls should or should not be allowed to compete in sports with other women and girls at each of the following levels?
a. Professional sports
b. College sports
c. High school sports
d. Youth sports

RESPONSE CATEGORIES
a. Should be allowed
b. Should not be allowed
c. No opinion

We provide statements about transgender youth. Please tell us how much you agree or disagree with each one.
a. State laws should ban transgender girls from competing against other girls in youth sports.
b. State laws should ban transgender women from competing against other women in college sports.

RESPONSE CATEGORIES
a. Strongly agree
b. Somewhat agree
c. Neither agree nor disagree
d. Somewhat disagree
e. Strongly disagree

We provide a few statements about transgender youth. Please tell us how much you agree or disagree with each one.
a. Excluding transgender kids from K-12 sports is discrimination.
b. If transgender girls are allowed to compete with other girls in youth sports, they would have a competitive advantage over other girls.
c. If transgender girls are allowed to compete with other girls in high school sports, they would have a competitive advantage over other girls.
d. If transgender boys are allowed to compete with other boys in youth sports, they would have a competitive advantage over other boys.
e. If transgender boys are allowed to compete with other boys in high school sports, they would have a competitive advantage over other boys.
f. Banning transgender girls from girls' sports will harm transgender girls.
g. I need more information to determine whether transgender girls should be allowed to compete with other girls in high school sports.
h. We should find a compromise between total bans on transgender girls participating in girls' sports versus allowing all transgender girls to compete in girls' sports.
i. There should be case-by case decisions about whether transgender girls can compete with other girls in sports.
j. There should be case-by case decisions about whether transgender boys can compete with other boys in sports.

RESPONSE CATEGORIES
a. Strongly agree
b. Somewhat agree
c. Neither agree nor disagree
d. Somewhat disagree
e. Strongly disagree

PCA Eigenvalues:

Comp1	7.62621
Comp2	3.88345
Comp3	1.67773
Comp4	1.16755
Comp5	.847625
Comp6	.612074
Comp7	.500284
Comp8	.444445

Comp9	.432971
Comp10	.370722
Comp11	.355769
Comp12	.337653
Comp13	.158068
Comp14	.126937
Comp15	.116866
Comp16	.10458
Comp17	.0861265
Comp18	.0797425
Comp19	.0712052

Appendix B: Pre-Registered Regression Results

	(1)	(2)	(3)	(4)
	Sex at Birth Policy	Sex at Birth Policy	Transgender Youth in Sport Scale	Transgender Youth in Sport Scale
Frame Condition				
Loss	0.06 (0.04)	0.05 (0.05)	0.04 (0.03)	0.04 (0.04)
Egalitarian	0.08 (0.04)*	0.06 (0.05)	0.08 (0.03)*	0.06 (0.04)
Acknowledge Discomfort	-0.00 (0.03)	-0.03 (0.05)	0.02 (0.02)	0.006 (0.04)
Loss * Acknowledge Discomfort	--	0.02 (0.07)	--	-0.002 (0.06)
Egal * Acknowledge Discomfort	--	0.06 (0.07)	--	0.04 (0.06)
Age	-0.004 (0.001)*	-0.004 (0.001)*	--	--
Partisanship	-0.05 (0.009)*	-0.05 (0.009)*	-0.10 (0.008)*	-0.10 (0.008)*
Egalitarianism	-0.20 (0.02)*	-0.20 (0.02)*	-0.14 (0.02)*	-0.14 (0.02)*
Disgust Sensitivity	-0.06 (0.02)*	-0.07 (0.02)*	--	--
Gender Roles	0.10 (0.02)*	0.10 (0.02)*	--	--
Traditionalism	0.27 (0.02)*	0.27 (0.02)*	0.35 (0.02)*	0.35 (0.02)*
Church Attendance	-0.05 (0.01)*	-0.05 (0.01)*	--	--
Born Again	-0.07 (0.04)	-0.07 (0.04)	--	--
Sports Fan	-0.03 (0.005)*	-0.03 (0.005)*	-0.02 (0.004)*	-0.02 (0.004)*
Female	--	--	0.07 (0.03)*	0.07 (0.03)*
Comfort with Transgender People	--	--	0.15 (0.02)*	0.15 (0.02)*
Ideology	--	--	-0.02 (0.008)*	-0.02 (0.008)*
Intercept	2.68 (0.07)*	2.69 (0.08)*	-0.05 (0.06)	-0.04 (0.06)
N	3,691	3,691	3,593	3,593
R-squared	0.29	0.29	0.46	0.46
σ	0.93	0.93	0.74	0.74

Appendix C: Regression results from alternative (not pre-registered) models

	(5)	(6)
	Ban Transgender Girls	Ban Transgender Boys
Frame Condition		
Loss	0.09 (0.05)	0.07 (0.05)
Egalitarian	0.18 (0.05)*	0.17 (0.05)*
Acknowledge Discomfort	-0.02 (0.04)	-0.006 (0.04)
Intercept	2.33 (0.03)*	2.41 (0.04)*
N	3,687	3,683
F (df, df)	5.02 (3, 3683)*	4.61 (3, 3679)*
R-squared	0.004	0.004
σ	1.17	1.15

	(7)	(8)	(9)	(10)	(11)
	Sex at Birth Policy	Sex at Birth Policy	Sex at Birth Policy	Transgender Youth in Sport Scale	Transgender Youth in Sport Scale
Frame Condition					
Loss	0.08 (0.07)	0.06 (0.04)	0.02 (0.4)	0.04 (0.03)	0.03 (0.04)
Egalitarian	-0.05 (0.07)	0.09 (0.04)*	0.05 (0.04)	0.08 (0.03)*	0.07 (0.03)
Acknowledge Discomfort	-0.004 (0.03)	-0.004 (0.03)	-0.002 (0.03)	0.02 (0.02)	0.02 (0.02)
Sports Fan	-0.03 (0.008)*	-0.03 (0.005)*	-0.03 (0.005)*	-0.02 (0.004)*	-0.01 (0.004)*
Sports Fan * Loss Frame	-0.004 (0.01)	--	--	--	--
Sports Fan * Egalitarian Frame	0.02 (0.01)*	--	--	--	--
Disgust Sensitivity	-0.07 (0.02)	-0.11 (0.03)	-0.07 (0.02)	--	--
Disgust * Loss Frame	--	0.04 (0.04)	--	--	--
Disgust * Egalitarian Frame	--	0.08 (0.04)*	--	--	--
Race/Ethnicity					
Black	--	--	-0.25	--	-0.26

			(0.10)*		(0.09)*
Latino	--	--	0.07 (0.09)	--	0.06 (0.07)
Other	--	--	0.009 (0.09)	--	0.002 (0.08)
Black * Loss Frame	--	--	0.36 (0.14)*	--	0.08 (0.12)
Latino * Loss Frame	--	--	0.04 (0.14)	--	-0.04 (0.10)
Other * Loss Frame	--	--	0.04 (0.13)	--	0.03 (0.11)
Black * Egalitarian Frame	--	--	0.38 (0.14)*	--	0.24 (0.12)*
Latino * Egalitarian Frame	--	--	-0.08 (0.14)	--	-0.13 (0.10)
Other * Egalitarian Frame	--	--	0.07 (0.13)	--	0.05 (0.10)
Egalitarianism	0.19 (0.02)*	0.20 (0.02)	0.20 (0.02)	0.15 (0.02)*	0.14 (0.02)*
Egalitarianism * Loss Frame	--	--	--	-0.02 (0.03)	--
Egalitarianism * Egalitarian Frame	--	--	--	-0.02 (0.03)	--
Age	-0.004 (0.001)*	-0.004 (0.001)*	-0.004 (0.001)*	--	--
Partisanship	-0.05 (0.01)*	-0.05 (0.01)*	-0.04 (0.01)*	-0.10 (0.008)*	-0.10 (0.008)*
Gender Roles	-0.10 (0.02)*	-0.10 (0.02)	-0.10 (0.02)	--	--
Traditionalism	-0.27 (0.02)	-0.27 (0.02)	-0.27 (0.02)	-0.35 (0.02)*	-0.35 (0.02)*
Church Attendance	-0.05 (0.04)	-0.05 (0.01)*	-0.05 (0.01)*	--	--
Born Again	-0.07 (0.04)	-0.07 (0.04)	-0.07 (0.04)	--	--
Female	--	--	--	0.07 (0.03)*	0.07 (0.03)*
Comfort with Transgender People	--	--	--	0.15 (0.02)*	0.15 (0.02)*
Ideology	--	--	--	-0.02 (0.008)*	-0.02 (0.008)*
Intercept	2.72 (0.08)*	2.68 (0.07)*	2.68 (0.08)*	-0.05 (0.06)	-0.02 (0.06)
N	3,691	3,691	3,691	3,593	3,593
R-squared	0.29	0.29	0.29	0.46	0.46

σ	0.93	0.93	0.93	0.74	0.73

Appendix D

We present some results that were not preregistered but offered some interesting findings. First, we present framing effect toplines for the policy questions about "banning" transgender boys and girls. Bans may be seen more like a discriminatory policy than a preference that requires transgender youth to participate in athletics based on their assigned sex at birth, so the egalitarian frame may have more pronounced effects on these attitudes. Second, we present heterogeneous treatment effects for characteristics that were identified by a causal forest model. Since these analyses were not preregistered, we consider these results as elucidating some interesting patterns in our data and can generate avenues for future work.

In Table 4 we present opinions on bans on transgender boys and girls by framing condition. Consistent with the previous results, we find that the egalitarian frame condition strongly related to opinions becoming less favorable to banning transgender boys and girls. Indeed, a majority of those in the control condition strongly or somewhat agreed with these bans. However, this flips for the egalitarian condition where a majority somewhat or strongly disagrees with bans on transgender girls (52.1%) and transgender boys (55.5%). The ATEs of the egalitarian frame are substantively larger on bans than our first policy dependent variable that asked about requiring transgender youth to only compete in sports based on their sex at birth (girls: $b = 0.18$, $se = 0.05$, $p < .001$; boys: $b = 0.17$, $se = 0.05$, $p < .001$).

Table 4: Responses to sports bans by framing condition

	Bans Transgender Girls			Bans on Transgender Boys		
	Control	Loss	Egalitarian	Control	Loss	Egalitarian
	% (*n*)	% (*n*)	% (*n*)	% (*n*)	% (*n*)	% (*n*)
Strongly agree	34.9% (430)	32.2% (395)	29.1% (357)	31.0% (381)	28.9% (354)	26.1% (320)
Somewhat agree	19.7% (243)	19.7% (242)	18.9% (232)	20.3% (250)	20.3% (249)	18.4% (226)
Somewhat disagree	23.7% (292)	22.8% (280)	25.0% (307)	25.8% (317)	25.0% (307)	27.1% (332)

Strongly disagree	21.6% (266)	25.3% (310)	27.1% (333)	22.9% (282)	25.8% (316)	28.4% (349)
N	1,231	1,227	1,229	1,230	1,226	1,227
χ^2 *(df)*	16.0 (6)*			14.9 (6)*		

Note: * $p < .05$.

Figure 2 plots heterogenous treatment effects on opinions opposing requiring transgender athletes to compete based on their sex at birth. The random forest model suggested that sports fandom and race/ethnicity yielded heterogeneous findings. Figure 2a suggests that the egalitarian frame was more effective among stronger sports fans than those who are less interested in sports. This is largely because stronger sports fans tend to hold more negative attitudes to transgender athletes (Flores 2020), and there is a negative relationship between fandom and policy opinions in the control group. The treatment appears to mute that negative correlation, which is why treatment effects are positive and significant as sports fandom increases. A similar pattern is observed by disgust sensitivity, where the egalitarian treatment is significantly positive among those higher in disgust sensitivity (Figure 2b). Treatment effects also appear markedly more profound among Black respondents relative to other racial and ethnic groups (Figure 2c). Similar to sports fans, Black respondents in the control group are significantly more favorable of this policy, and the pattern is muted in either frame condition.

Figure 2: Heterogenous treatment effects on opinions opposing requiring transgender athletes to compete based on their sex at birth for (a) sports fandom, (b) disgust sensitivity, and (c) race/ethnicity

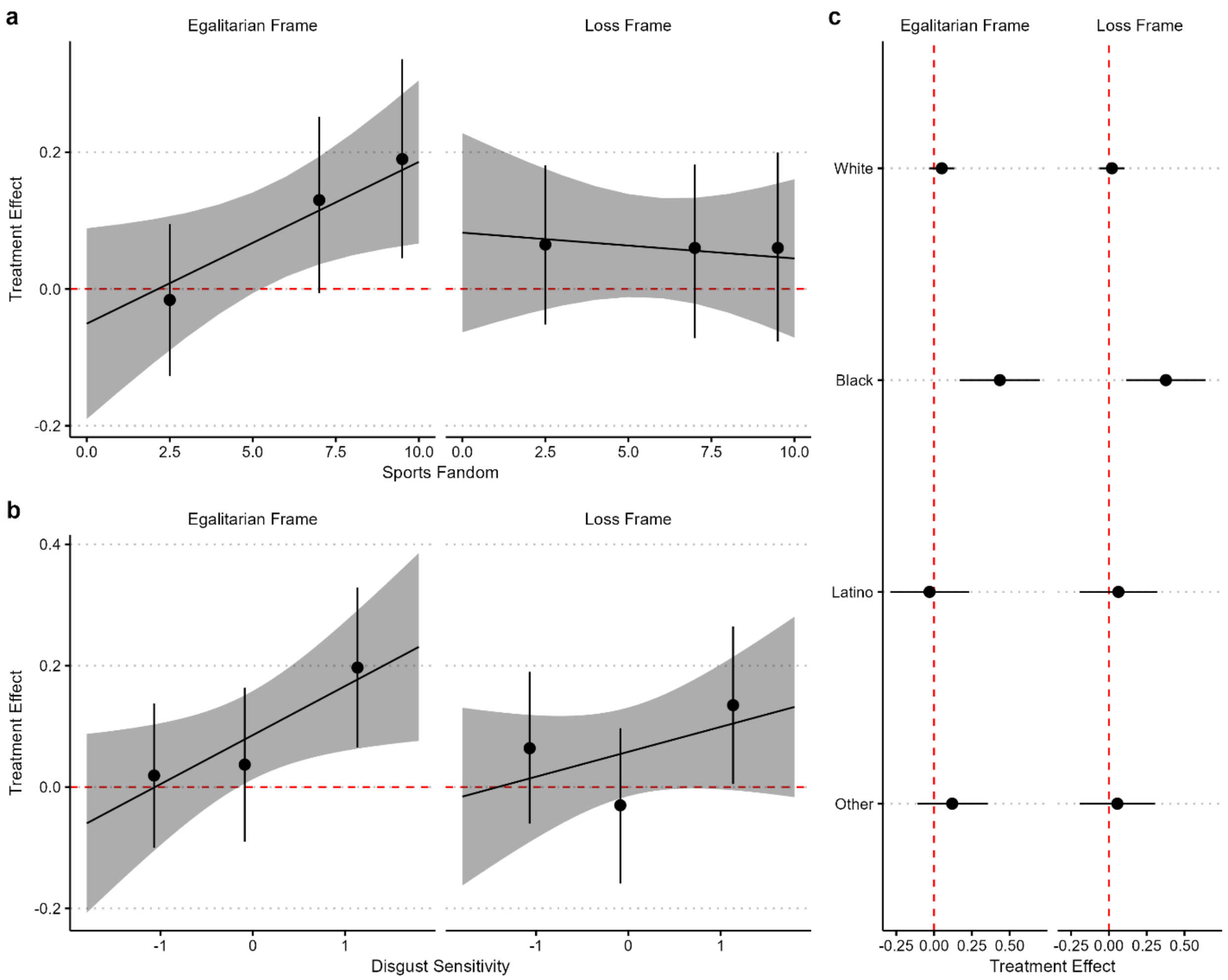

Note: 95% confidence intervals represented by shaded region or line segments; sports fandom was discretized to assess linearity assumptions.

Figure 3 plots heterogenous treatment effects on the transgender sports scale. The random forest model suggested that egalitarianism and race/ethnicity resulted in significant differences. Figure 3a suggests that treatment effects of the egalitarian frame differed at various levels of egalitarianism. For those high in egalitarianism, there was no significant effect of the egalitarian frame. This is likely due to high egalitarians having more supportive opinions on transgender rights that emphasizing their egalitarian values reaches a ceiling effect. Whereas, the effect treatment effect appeared to be most impactful among those who score in the middle of the egalitarianism scale. This suggests that those who hold a mix of egalitarian and inegalitarian

views may be more persuaded by the egalitarian frame. The confidence intervals and treatment effects appear to slightly decline among low egalitarians.[1] Figure 3b suggests the egalitarian frame was particularly effective among Black respondents. However and unlike Figure 2b, the loss frame does not appear to have a significant effect among Black respondents. This may suggest that while both frames were immediately effective among Black respondents, the egalitarian frame was strong enough to persist across numerous items in the post-test.

Figure 3: Heterogenous treatments effects in the transgender sports scale by (a) egalitarianism and (b) race/ethnicity

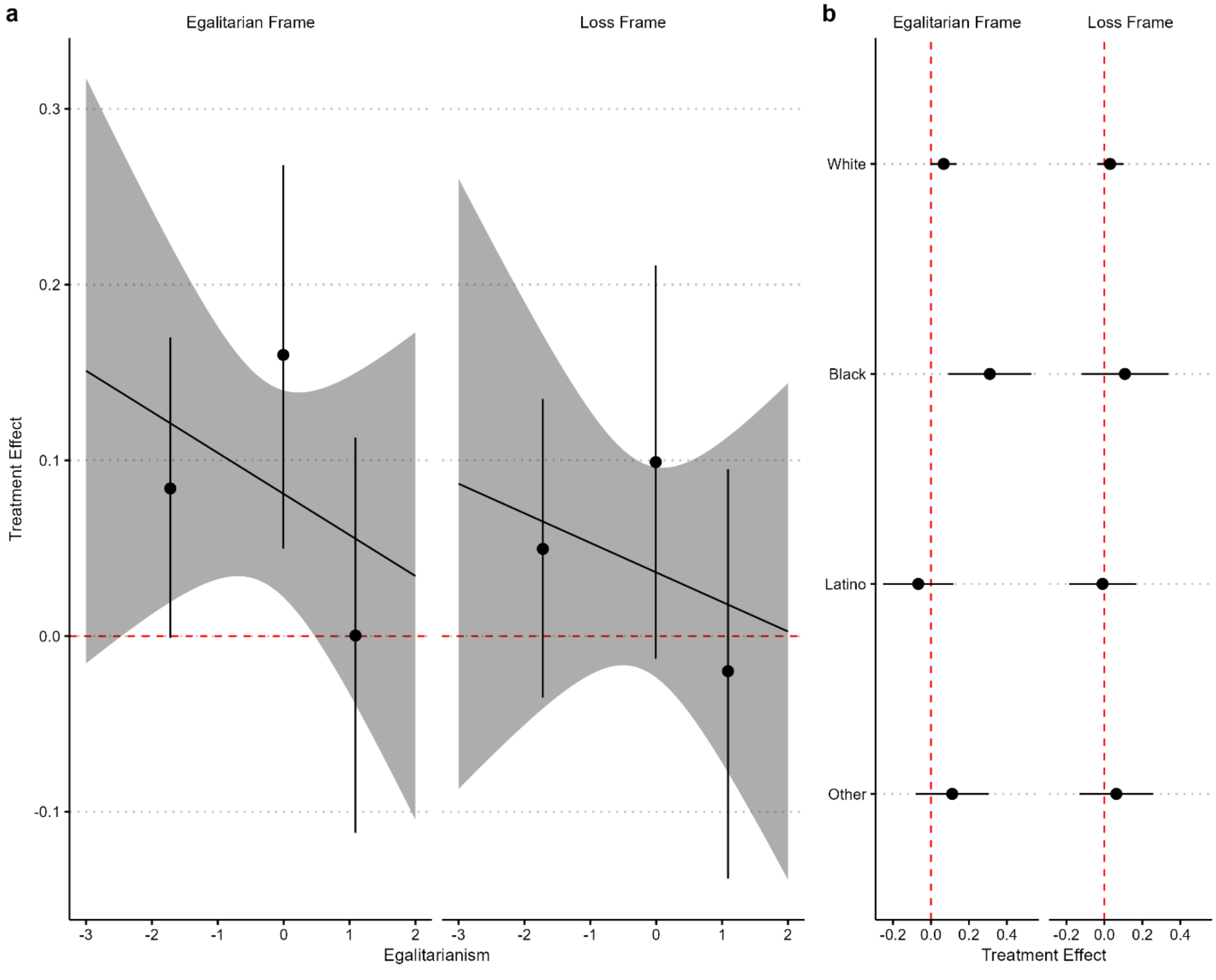

Note: 95% confidence intervals represented by shaded region or line segments; sports fandom was discretized to assess linearity assumptions.

[1] While model prediction in Figure 2a are from a linear model, a curvilinear model shows these patterns more clearly.
